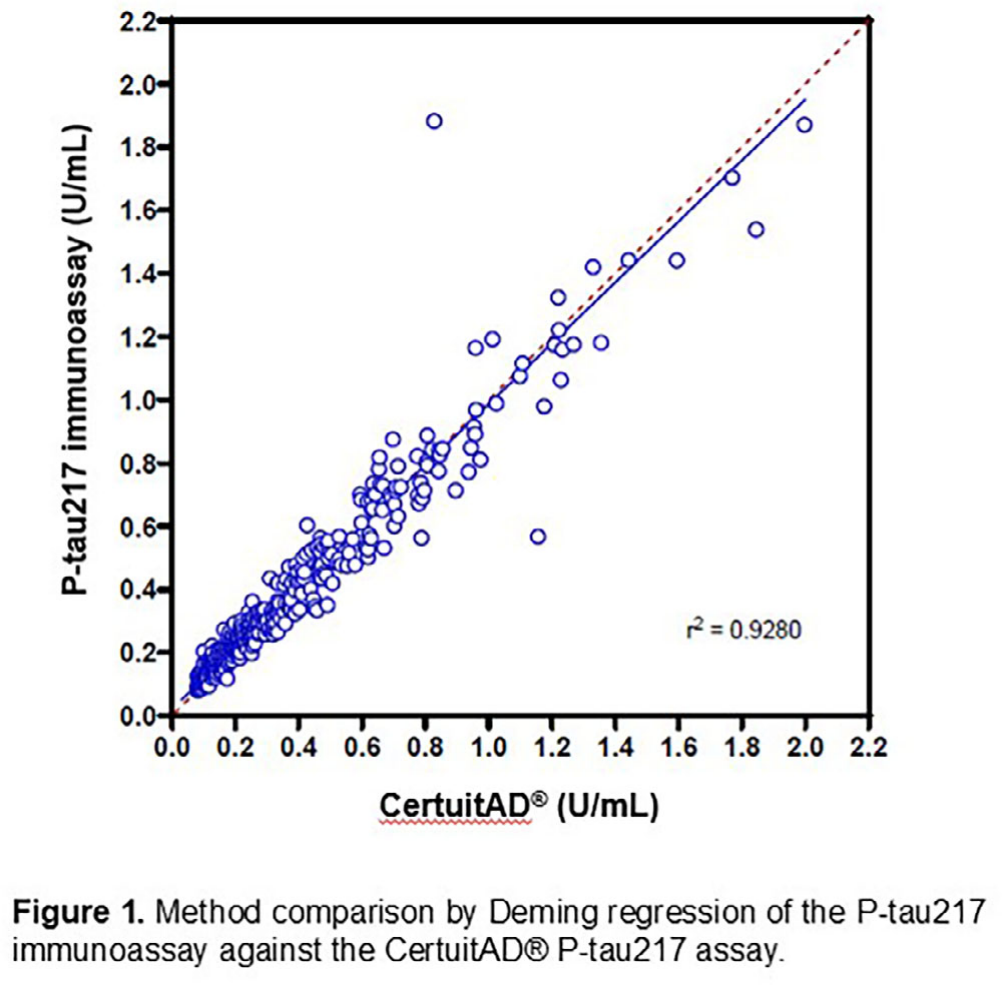# Analytical validation of plasma *p* ‐tau217 on the SP‐X platform

**DOI:** 10.1002/alz70861_108797

**Published:** 2025-12-23

**Authors:** Heather A Nelson, J Alan Erickson, Sonia L La’ulu, Sierra Cunningham, Kelly Doyle

**Affiliations:** ^1^ ARUP Institute for Clinical and Experimental Pathology, Salt Lake City, UT USA; ^2^ University of Utah Health, Salt Lake City, UT USA; ^3^ ARUP Institute for Research and Innovation in Diagnostic and Precision Medicine, Salt Lake City, UT USA

## Abstract

**Background:**

Blood‐based biomarkers, specifically plasma *p* ‐tau217, have emerged as diagnostic biomarkers in the workup of Alzheimer disease (AD). Here we have validated a chemiluminescent immunoassay for plasma *p* ‐tau217.

**Methods:**

Plasma *p* ‐tau217 was measured using the Quanterix SP‐X Human *p* ‐tau217 immunoassay kit on two SP‐X imagers following the kit manufacturer’s protocol. Tested specimens included plasma from a cohort of patients presenting with cognitive decline and CSF spiked plasma. Performance characteristics evaluated included a method comparison against the CertuitAD® *p* ‐tau217 assay (Eli Lilly Clinical Diagnostics Laboratory), analyte recovery, precision, analytical sensitivity, analyte stability, and interference/cross‐reactivity. Reference cut‐points for classifying disease status were also established.

**Results:**

A method comparison study against CertuitAD® assay generated a slope of 0.965, intercept of 0.021, r^2^ of 0.928 and bias of 6.0% (*n* =396, Deming regression, Figure 1). Percent recoveries across the analytical measuring range varied between 87.3–114.8%. Within‐run precision across two imagers had CVs ranging from 3.3–6.3, 3.5–5.8 and 5.1–9.6% for high, mid and low *p* ‐tau217 concentrations, respectively. Within‐laboratory CVs were 4.4–8.8, 4.8–6.9 and 9.3–12.4% for high, mid and low concentrations, respectively. The limit of detection was 0.04 U/mL. The limit of quantitation was established at 0.70 U/mL. *p* ‐tau217 protein stabilities were determined for minimums of 48 hours, 7 days, 2 weeks and 3 cycles for ambient, refrigerated, frozen (‐20 °C) and freeze/thaw, respectively. Interference (outside ±20% from matrix‐matched controls) was evident above 300 and 4.3 mg/dL for hemoglobin and bilirubin, respectively. Lipemia (1500 mg/dL), rheumatoid factor (99 IU/mL), a 47‐analyte drug panel, *p* ‐tau181 (10 pg/mL), and human tau441 (20ng/mL) did not interfere with the assay. Analysis of positron emission tomography combined with *p* ‐tau217 results from individuals presenting with cognitive decline (*n* =524, ages 60–86 years), established cut‐offs of Negative, <0.13 U/mL; Indeterminate, ≥0.13 to <0.20 U/mL; and Positive, ≥0.20 U/mL.

**Conclusions:**

The Quanterix SP‐X Human *p* ‐tau217 immunoassay demonstrates acceptable performance for quantifying *p* ‐tau217 in human plasma. Results support the assay as an aid in the detection of AD pathology.